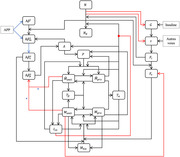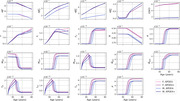# A nano‐ to microscale, complex and multifactorial computational model of Alzheimer’s disease

**DOI:** 10.1002/alz.088850

**Published:** 2025-01-03

**Authors:** Simon Duchesne, Éléonore Chamberland, Seyedadel Moravveji, Nicolas Doyon

**Affiliations:** ^1^ Centre de recherche de l’Institut Universitaire de Cardiologie et de Pneumologie de Québec, Quebec, QC Canada; ^2^ Radiology and Nuclear Medicine Department, University Laval, Quebec City, QC Canada; ^3^ Université Laval, Quebec City, QC Canada; ^4^ CERVO brain research centre, Quebec, QC Canada; ^5^ Université Laval, Quebec city, QC Canada

## Abstract

**Background:**

While individual etiological hypotheses for AD are researched, few large‐scale theoretical integrative efforts linking entities involved in these dysfunctions have been attempted. Experimentally, assessing such a global theory is logistically near impossible to achieve as the number of variables is substantial. Alternatively, computational neuroscience allows for the joint study of multiple entities at this scale, the generation of predictions, and their validation with real data. In this work, we present a theoretical computational model describing the progression of AD through an individual’s lifespan covering more than 50 different entities.

**Method:**

Our computational model is a system of 19 ordinary differential equations (*cf*. Figure 1). The equations describe the evolution of proteins at the nanoscale (Aβ monomers, oligomers and plaques; tau filaments and tangles, anti‐inflammatory cytokines, insulin) and cell populations at the microscale (neurons, astrocytes, macrophages, microglia). Equations were parametrized according to sex and APOE status, and initial conditions chosen from the literature. Our key outcomes of interest were the accumulation of recognized pathological markers of AD (Aβ monomers or plaques, tau filaments or tangles) and neuronal death. The model was solved in daily increments over a 50‐year lifespan.

**Results:**

The evolution of every variable of our model is shown in Figure 2. For the different forms of amyloid, the curves for APOE4‐negative men and women mostly overlap. This is also observed for APOE4‐positive individuals. However, it is important to note that despite the overlap, there are noticeable variations in the curves between different groups. Neuronal loss occurs earlier in individuals with an APOE4 allele regardless of sex. We obtain losses of 10.9% and 11.7%, for APOE4‐ and APOE+ women respectively, and of 10.9% and 12.1% for APOE4‐ and APOE4+ men respectively, which are commensurate with the literature. We also observe a transition to a proinflammatory state occurring around 50 years of age, with women who are APOE4+ experience this transition earlier, followed by APOE4+ men, APOE4‐ women, and finally APOE4‐ men.

**Conclusion:**

Computational models represent an essential initial step toward constructing a complex, predictive framework, and hold significant potential for identifying effective therapeutic targets in the fight against AD.